# Supplementary material for: The out-of-field dose in radiation therapy induces delayed tumorigenesis by senescence evasion
Source: eLife. 2022 Mar 18;11:e67190. doi: 10.7554/eLife.67190 (PMC8933005; doi:10.7554/eLife.67190)
Supplement: Figure 2—figure supplement 4—source data 1. [file elife-67190-fig2-figsupp4-data1.pptx]

## Slide 1
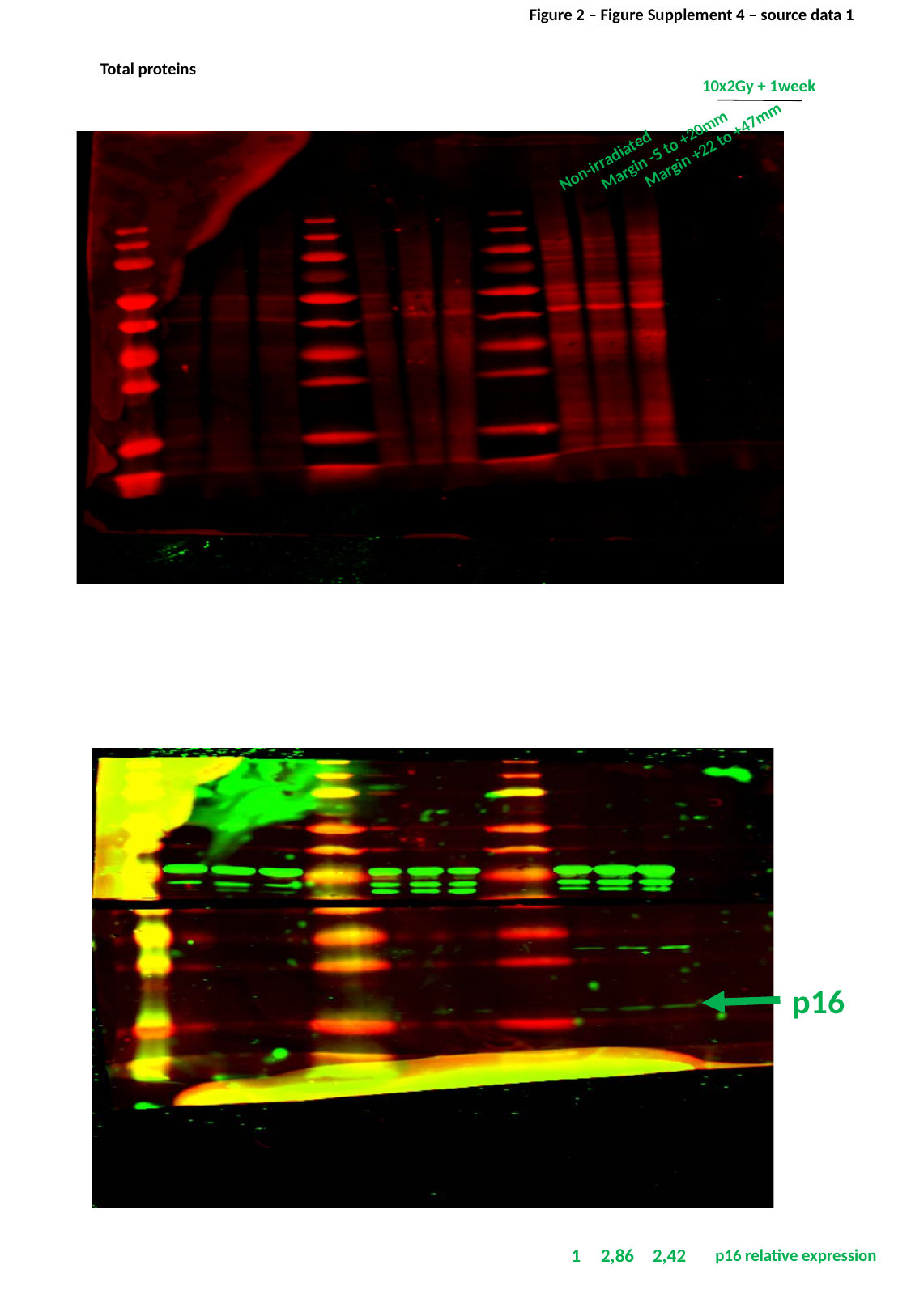

Figure 2 – Figure Supplement 4 – source data 1
Total proteins
10x2Gy + 1week
Margin +22 to +47mm
Margin -5 to +20mm
Non-irradiated
p16
2,86
1
2,42
p16 relative expression
