# Supplementary material for: The out-of-field dose in radiation therapy induces delayed tumorigenesis by senescence evasion
Source: eLife. 2022 Mar 18;11:e67190. doi: 10.7554/eLife.67190 (PMC8933005; doi:10.7554/eLife.67190)
Supplement: Figure 3—source data 3. [file elife-67190-fig3-data3.pdf]

Col. stats		A	B	C	D
		Non-irradiated	PTV	-5 to +20	+22 to +47
		Y	Y	Y	Y
1	Number of values	35	40	44	45
2					
3	Minimum	0.0	0.0	0.2985	0.0
4	25% Percentile	0.0	97.64	52.76	56.98
5	Median	0.0	114.8	70.49	69.88
6	75% Percentile	69.64	136.8	87.72	80.09
7	Maximum	109.6	179.8	111.5	120.8
8					
9	Mean	31.23	114.4	68.20	65.95
10	Std. Deviation	39.75	34.19	26.61	28.76
11	Std. Error of Mean	6.719	5.405	4.012	4.288
12					
13	Lower 95% CI of mean	17.58	103.4	60.11	57.31
14	Upper 95% CI of mean	44.89	125.3	76.29	74.59
15					
16	D'Agostino & Pearson omnibus normality test				
17	K2	19.48	24.48	6.362	8.983
18	P value	< 0.0001	< 0.0001	0.0415	0.0112
19	Passed normality test (alpha=0.05)?	No	No	No	No
20	P value summary	****	****	*	*
21					
22	Sum	1093	4574	3001	2968

1way ANOVA<br>ANOVA		
1	Table Analyzed	temps 0h pH12 F6
2		
3	Kruskal-Wallis test	
4	P value	< 0.0001
5	Exact or approximate P value?	Approximate
6	P value summary	****
7	Do the medians vary signif. (P < 0.05)	Yes
8	Number of groups	4
9	Kruskal-Wallis statistic	74.91
10		
11	Data summary	
12	Number of treatments (columns)	4
13	Number of values (total)	164

1way ANOVA<br>Multiple comparisons						
1	Number of families	1				
2	Number of comparisons per family	3				
3	Alpha	0.05				
4						
5	Dunn's multiple comparisons test	Mean rank diff.	Significant?	Summary		
6						
7	Non-irradiated vs. PTV	-91.06	Yes	****		
8	Non-irradiated vs. -5 to +20	-32.52	Yes	**		
9	Non-irradiated vs. +22 to +47	-28.88	Yes	*		
10						
11						
12	Test details	Mean rank 1	Mean rank 2	Mean rank diff.	n1	n2
13						
14	Non-irradiated vs. PTV	43.64	134.7	-91.06	35	40
15	Non-irradiated vs. -5 to +20	43.64	76.16	-32.52	35	44
16	Non-irradiated vs. +22 to +47	43.64	72.52	-28.88	35	45
