# Supplementary material for: The out-of-field dose in radiation therapy induces delayed tumorigenesis by senescence evasion
Source: eLife. 2022 Mar 18;11:e67190. doi: 10.7554/eLife.67190 (PMC8933005; doi:10.7554/eLife.67190)
Supplement: Figure 3—source data 6. [file elife-67190-fig3-data6.pdf]

Col. stats		A	B	C	D
		Non-irradiated	PTV	-5 to +20	+22 to +47
		Y	Y	Y	Y
1	Number of values	83	126	99	131
2					
3	Minimum	0.0	0.0	0.0	0.0
4	25% Percentile	0.0	0.0	0.0	0.0
5	Median	0.0	1.149	0.0	0.0
6	75% Percentile	3.913	10.39	0.0	3.409
7	Maximum	32.62	71.89	69.23	61.01
8					
9	Mean	2.346	7.497	2.968	3.454
10	Std. Deviation	5.155	14.51	9.077	8.927
11	Std. Error of Mean	0.5659	1.293	0.9123	0.7800
12					
13	Lower 95% CI of mean	1.221	4.938	1.157	1.911
14	Upper 95% CI of mean	3.472	10.06	4.778	4.997
15					
16	D'Agostino & Pearson omnibus normality test				
17	K2	87.37	93.34	135.0	144.1
18	P value	< 0.0001	< 0.0001	< 0.0001	< 0.0001
19	Passed normality test (alpha=0.05)?	No	No	No	No
20	P value summary	****	****	****	****
21					
22	Sum	194.7	944.6	293.8	452.4

1way ANOVA
ANOVA

1	Table Analyzed	temps 0h pH8 F6
2		
3	Kruskal-Wallis test	
4	P value	< 0.0001
5	Exact or approximate P value?	Approximate
6	P value summary	****
7	Do the medians vary signif. (P < 0.05)	Yes
8	Number of groups	4
9	Kruskal-Wallis statistic	22.34
10		
11	Data summary	
12	Number of treatments (columns)	4
13	Number of values (total)	439

1way ANOVA
Multiple comparisons

1	Number of families	1				
2	Number of comparisons per family	3				
3	Alpha	0.05				
4						
5	Dunn's multiple comparisons test	Mean rank diff.	Significant?	Summary		
6						
7	Non-irradiated vs. PTV	-51.92	Yes	**		
8	Non-irradiated vs. -5 to +20	12.05	No	ns		
9	Non-irradiated vs. +22 to +47	-13.99	No	ns		
10						
11						
12	Test details	Mean rank 1	Mean rank 2	Mean rank diff.	n1	n2
13						
14	Non-irradiated vs. PTV	203.6	255.6	-51.92	83	126
15	Non-irradiated vs. -5 to +20	203.6	191.6	12.05	83	99
16	Non-irradiated vs. +22 to +47	203.6	217.6	-13.99	83	131
