# Supplementary material for: The out-of-field dose in radiation therapy induces delayed tumorigenesis by senescence evasion
Source: eLife. 2022 Mar 18;11:e67190. doi: 10.7554/eLife.67190 (PMC8933005; doi:10.7554/eLife.67190)
Supplement: Figure 3—figure supplement 3—source data 2. [file elife-67190-fig3-figsupp3-data2.pdf]

Col. stats		A	B	C	D
		Non-irradiated	PTV	-5 to +20	+22 to +47
		Y	Y	Y	Y
1	Number of values	87	68	84	87
2					
3	Minimum	0.0	88.70	0.0	0.0
4	25% Percentile	84.67	142.4	139.2	109.7
5	Median	118.2	166.1	155.4	153.0
6	75% Percentile	164.3	196.1	190.7	199.3
7	Maximum	211.3	276.7	271.7	370.7
8					
9	Mean	122.2	168.1	163.2	164.5
10	Std. Deviation	47.16	38.61	41.72	73.18
11	Std. Error of Mean	5.056	4.683	4.552	7.846
12					
13	Lower 95% CI of mean	112.1	158.8	154.1	148.9
14	Upper 95% CI of mean	132.2	177.5	172.3	180.1
15					
16	D'Agostino & Pearson omnibus normality test				
17	K2	0.7440	0.9166	7.871	7.447
18	P value	0.6893	0.6323	0.0195	0.0242
19	Passed normality test (alpha=0.05)?	Yes	Yes	No	No
20	P value summary	ns	ns	*	*
21					
22	Sum	10629	11433	13709	14313

1way ANOVA
ANOVA

1	Table Analyzed	F27FC1 pH12.3
2		
3	Kruskal-Wallis test	
4	P value	< 0.0001
5	Exact or approximate P value?	Approximate
6	P value summary	****
7	Do the medians vary signif. (P < 0.05)	Yes
8	Number of groups	4
9	Kruskal-Wallis statistic	40.35
10		
11	Data summary	
12	Number of treatments (columns)	4
13	Number of values (total)	326

1way ANOVA
Multiple comparisons

1	Number of families	1				
2	Number of comparisons per family	3				
3	Alpha	0.05				
4						
5	Dunn's multiple comparisons test	Mean rank diff.	Significant?	Summary		
6						
7	Non-irradiated vs. PTV	-84.81	Yes	****		
8	Non-irradiated vs. -5 to +20	-74.92	Yes	****		
9	Non-irradiated vs. +22 to +47	-60.49	Yes	****		
10						
11						
12	Test details	Mean rank 1	Mean rank 2	Mean rank diff.	n1	n2
13						
14	Non-irradiated vs. PTV	110.4	195.2	-84.81	87	68
15	Non-irradiated vs. -5 to +20	110.4	185.3	-74.92	87	84
16	Non-irradiated vs. +22 to +47	110.4	170.9	-60.49	87	87
