# Supplementary material for: The out-of-field dose in radiation therapy induces delayed tumorigenesis by senescence evasion
Source: eLife. 2022 Mar 18;11:e67190. doi: 10.7554/eLife.67190 (PMC8933005; doi:10.7554/eLife.67190)
Supplement: Figure 3—figure supplement 3—source data 4. [file elife-67190-fig3-figsupp3-data4.pdf]

Col. stats		A	B	C	D
		Non-irradiated	PTV	-5 to +20	+22 to +47
		Y	Y	Y	Y
1	Number of values	181	151	205	209
2					
3	Minimum	0.0	0.0	0.0	0.0
4	25% Percentile	0.0	0.0	0.0	0.0
5	Median	0.0	0.0	0.0	0.0
6	75% Percentile	0.0	55.88	0.0	0.0
7	Maximum	162.1	321.7	136.4	102.4
8					
9	Mean	13.62	31.02	6.625	6.092
10	Std. Deviation	33.00	54.84	20.26	19.58
11	Std. Error of Mean	2.453	4.463	1.415	1.355
12					
13	Lower 95% CI of mean	8.785	22.20	3.836	3.421
14	Upper 95% CI of mean	18.46	39.84	9.415	8.762
15					
16	D'Agostino & Pearson omnibus normality test				
17	K2	100.2	107.7	182.7	156.2
18	P value	< 0.0001	< 0.0001	< 0.0001	< 0.0001
19	Passed normality test (alpha=0.05)?	No	No	No	No
20	P value summary	****	****	****	****
21					
22	Sum	2466	4684	1358	1273

1way ANOVA
ANOVA

1	Table Analyzed	F27FC1 pH8
2		
3	Kruskal-Wallis test	
4	P value	< 0.0001
5	Exact or approximate P value?	Approximate
6	P value summary	****
7	Do the medians vary signif. (P < 0.05)	Yes
8	Number of groups	4
9	Kruskal-Wallis statistic	52.26
10		
11	Data summary	
12	Number of treatments (columns)	4
13	Number of values (total)	746

**1way ANOVA**<br>Multiple comparisons						
**1**	Number of families	1				
**2**	Number of comparisons per family	3				
**3**	Alpha	0.05				
**4**						
**5**	Dunn's multiple comparisons test	Mean rank diff.	Significant?	Summary		
**6**						
**7**	Non-irradiated vs. PTV	-71.12	Yes	****		
**8**	Non-irradiated vs. -5 to +20	23.00	No	ns		
**9**	Non-irradiated vs. +22 to +47	28.92	No	ns		
**10**						
**11**						
**12**	Test details	Mean rank 1	Mean rank 2	Mean rank diff.	n1	n2
**13**						
**14**	Non-irradiated vs. PTV	373.5	444.7	-71.12	181	151
**15**	Non-irradiated vs. -5 to +20	373.5	350.5	23.00	181	205
**16**	Non-irradiated vs. +22 to +47	373.5	344.6	28.92	181	209
