# Supplementary material for: The out-of-field dose in radiation therapy induces delayed tumorigenesis by senescence evasion
Source: eLife. 2022 Mar 18;11:e67190. doi: 10.7554/eLife.67190 (PMC8933005; doi:10.7554/eLife.67190)
Supplement: Figure 3—figure supplement 3—source data 6. [file elife-67190-fig3-figsupp3-data6.pdf]

Col. stats		A	B	C	D
		Non-irradiated	PTV	-5 to +20	+22 to +47
		Y	Y	Y	Y
1	Number of values	50	50	62	78
2					
3	Minimum	0.0	0.0	0.0	0.0
4	25% Percentile	13.97	18.95	16.51	14.79
5	Median	21.22	26.89	28.73	24.22
6	75% Percentile	27.25	39.22	45.19	43.79
7	Maximum	51.33	141.7	168.4	170.7
8					
9	Mean	20.77	30.93	35.26	33.96
10	Std. Deviation	11.62	25.86	29.33	30.86
11	Std. Error of Mean	1.643	3.658	3.725	3.495
12					
13	Lower 95% CI of mean	17.46	23.58	27.81	27.00
14	Upper 95% CI of mean	24.07	38.28	42.71	40.92
15					
16	D'Agostino & Pearson omnibus normality test				
17	K2	1.393	39.71	40.74	47.97
18	P value	0.4983	< 0.0001	< 0.0001	< 0.0001
19	Passed normality test (alpha=0.05)?	Yes	No	No	No
20	P value summary	ns	****	****	****
21					
22	Sum	1038	1546	2186	2649

1way ANOVA<br>ANOVA		
1	Table Analyzed	temps 0h ph12 F1
2		
3	Kruskal-Wallis test	
4	P value	0.0306
5	Exact or approximate P value?	Approximate
6	P value summary	*
7	Do the medians vary signif. (P < 0.05)	Yes
8	Number of groups	4
9	Kruskal-Wallis statistic	8.904
10		
11	Data summary	
12	Number of treatments (columns)	4
13	Number of values (total)	240

1way ANOVA<br>Multiple comparisons						
1	Number of families	1				
2	Number of comparisons per family	3				
3	Alpha	0.05				
4						
5	Dunn's multiple comparisons test	Mean rank diff.	Significant?	Summary		
6						
7	Non-irradiated vs. PTV	-29.70	No	ns		
8	Non-irradiated vs. -5 to +20	-37.84	Yes	*		
9	Non-irradiated vs. +22 to +47	-27.10	No	ns		
10						
11						
12	Test details	Mean rank 1	Mean rank 2	Mean rank diff.	n1	n2
13						
14	Non-irradiated vs. PTV	95.73	125.4	-29.70	50	50
15	Non-irradiated vs. -5 to +20	95.73	133.6	-37.84	50	62
16	Non-irradiated vs. +22 to +47	95.73	122.8	-27.10	50	78
