# Supplementary material for: The out-of-field dose in radiation therapy induces delayed tumorigenesis by senescence evasion
Source: eLife. 2022 Mar 18;11:e67190. doi: 10.7554/eLife.67190 (PMC8933005; doi:10.7554/eLife.67190)
Supplement: Figure 3—figure supplement 3—source data 8. [file elife-67190-fig3-figsupp3-data8.pdf]

Col. stats		A	B	C	D
		Non-irradiated	PTV	-5 to +20	+22 to +47
		Y	Y	Y	Y
1	Number of values	59	77	61	84
2					
3	Minimum	0.003125	0.002329	0.009682	0.01231
4	25% Percentile	0.09735	0.4734	0.4008	0.1864
5	Median	0.4121	1.469	1.749	0.8709
6	75% Percentile	2.127	5.509	5.365	1.840
7	Maximum	26.87	135.7	103.2	156.3
8					
9	Mean	2.623	12.79	7.673	5.079
10	Std. Deviation	5.202	28.35	17.43	19.27
11	Std. Error of Mean	0.6772	3.231	2.231	2.102
12					
13	Lower 95% CI of mean	1.267	6.359	3.209	0.8975
14	Upper 95% CI of mean	3.978	19.23	12.14	9.260
15					
16	D'Agostino & Pearson omnibus normality test				
17	K2	56.58	65.90	79.51	147.4
18	P value	< 0.0001	< 0.0001	< 0.0001	< 0.0001
19	Passed normality test (alpha=0.05)?	No	No	No	No
20	P value summary	****	****	****	****
21					
22	Sum	154.7	985.1	468.1	426.6

1way ANOVA
ANOVA

1	Table Analyzed	temps 0h ph8 f1
2		
3	Kruskal-Wallis test	
4	P value	0.0004
5	Exact or approximate P value?	Approximate
6	P value summary	***
7	Do the medians vary signif. (P < 0.05)	Yes
8	Number of groups	4
9	Kruskal-Wallis statistic	18.37
10		
11	Data summary	
12	Number of treatments (columns)	4
13	Number of values (total)	281

**1way ANOVA**<br>Multiple comparisons						
**1**	Number of families	1				
**2**	Number of comparisons per family	3				
**3**	Alpha	0.05				
**4**						
**5**	Dunn's multiple comparisons test	Mean rank diff.	Significant?	Summary		
**6**						
**7**	Non-irradiated vs. PTV	-46.52	Yes	**		
**8**	Non-irradiated vs. -5 to +20	-51.86	Yes	**		
**9**	Non-irradiated vs. +22 to +47	-15.09	No	ns		
**10**						
**11**						
**12**	Test details	Mean rank 1	Mean rank 2	Mean rank diff.	n1	n2
**13**						
**14**	Non-irradiated vs. PTV	112.5	159.0	-46.52	59	77
**15**	Non-irradiated vs. -5 to +20	112.5	164.3	-51.86	59	61
**16**	Non-irradiated vs. +22 to +47	112.5	127.6	-15.09	59	84
