# Supplementary material for: The out-of-field dose in radiation therapy induces delayed tumorigenesis by senescence evasion
Source: eLife. 2022 Mar 18;11:e67190. doi: 10.7554/eLife.67190 (PMC8933005; doi:10.7554/eLife.67190)
Supplement: Figure 3—figure supplement 5—source data 2. [file elife-67190-fig3-figsupp5-data2.pdf]

Col. stats		A	B	C	D	E	F	G	H
		Non-irradiated	PTV	-5 to +20mm	+45 to +60mm	+87 to +112mm	+162 to +187mm	+87 to +112mm	+162 to +187mm
		Y	Y	Y	Y	Y	Y	Y	Y
1	Number of values	25	26	30	33	24	34	34	48
2									
3	Minimum	0.0	0.0	0.0	0.0	0.0	0.0	0.0	0.0
4	25% Percentile	0.0	4.000	2.000	1.500	1.000	2.000	0.0	0.0
5	Median	3.000	7.500	4.000	4.000	3.000	4.000	1.000	1.000
6	75% Percentile	4.000	14.75	7.250	15.00	12.75	10.25	2.500	4.000
7	Maximum	13.00	24.00	30.00	46.00	22.00	32.00	57.00	14.00
8									
9	Mean	3.240	9.654	5.867	9.727	6.875	7.412	3.235	2.458
10	Std. Deviation	3.503	7.099	6.917	12.94	7.291	8.528	9.838	3.017
11	Std. Error of Mean	0.7007	1.392	1.263	2.253	1.488	1.463	1.687	0.4355
12									
13	Lower 95% CI of mean	1.794	6.787	3.284	5.139	3.796	4.436	-0.1974	1.582
14	Upper 95% CI of mean	4.686	12.52	8.450	14.32	9.954	10.39	6.668	3.334
15									
16	D'Agostino & Pearson omnibus normality test								
17	K2	9.574	2.595	24.05	18.74	3.834	15.75	74.26	28.41
18	P value	0.0083	0.2732	< 0.0001	< 0.0001	0.1471	0.0004	< 0.0001	< 0.0001
19	Passed normality test (alpha=0.05)?	No	Yes	No	No	Yes	No	No	No
20	P value summary	**	ns	****	****	ns	***	****	****
21									
22	Sum	81.00	251.0	176.0	321.0	165.0	252.0	110.0	118.0

1way ANOVA<br>ANOVA		
1	Table Analyzed	XRCC1
2		
3	Kruskal-Wallis test	
4	P value	< 0.0001
5	Exact or approximate P value?	Approximate
6	P value summary	****
7	Do the medians vary signif. (P < 0.05)	Yes
8	Number of groups	8
9	Kruskal-Wallis statistic	47.32
10		
11	Data summary	
12	Number of treatments (columns)	8
13	Number of values (total)	254

**1way ANOVA** Multiple comparisons						
1	Number of families	1				
2	Number of comparisons per family	7				
3	Alpha	0.05				
4						
5	Dunn's multiple comparisons test	Mean rank diff.	Significant?	Summary		
6						
7	Non-irradiated vs. PTV	-70.82	Yes	**		
8	Non-irradiated vs. -5 to +20mm	-29.10	No	ns		
9	Non-irradiated vs. +45 to +60mm	-42.38	No	ns		
10	Non-irradiated vs. +87 to +112mm	-28.63	No	ns		
11	Non-irradiated vs. +162 to +187mm	-39.13	No	ns		
12	Non-irradiated vs. +87 to +112mm	30.47	No	ns		
13	Non-irradiated vs. +162 to +187mm	14.16	No	ns		
14						
15						
16	Test details	Mean rank 1	Mean rank 2	Mean rank diff.	n1	n2
17						
18	Non-irradiated vs. PTV	110.1	180.9	-70.82	25	26
19	Non-irradiated vs. -5 to +20mm	110.1	139.2	-29.10	25	30
20	Non-irradiated vs. +45 to +60mm	110.1	152.5	-42.38	25	33
21	Non-irradiated vs. +87 to +112mm	110.1	138.8	-28.63	25	24
22	Non-irradiated vs. +162 to +187mm	110.1	149.3	-39.13	25	34
23	Non-irradiated vs. +87 to +112mm	110.1	79.65	30.47	25	34
24	Non-irradiated vs. +162 to +187mm	110.1	95.96	14.16	25	48
