# Supplementary material for: The out-of-field dose in radiation therapy induces delayed tumorigenesis by senescence evasion
Source: eLife. 2022 Mar 18;11:e67190. doi: 10.7554/eLife.67190 (PMC8933005; doi:10.7554/eLife.67190)
Supplement: Figure 3—figure supplement 5—source data 4. [file elife-67190-fig3-figsupp5-data4.pdf]

Col. stats		A	B	C	D	E	F	G	H
		Non-irradiated	PTV	-5 to +20mm	+45 to +60mm	+87 to +112mm	+162 to +187mm	+87 to +112mm	+162 to +187mm
		Y	Y	Y	Y	Y	Y	Y	Y
1	Number of values	32	74	28	34	64	58	30	23
2									
3	Minimum	0.0	0.0	0.0	0.0	0.0	0.0	0.0	0.0
4	25% Percentile	0.0	0.0	1.250	0.0	0.2500	0.0	1.000	0.0
5	Median	0.0	7.000	3.000	1.500	1.000	1.000	2.000	1.000
6	75% Percentile	1.750	13.00	7.000	3.000	2.000	3.000	3.000	3.000
7	Maximum	10.00	34.00	20.00	18.00	6.000	22.00	15.00	7.000
8									
9	Mean	1.219	8.297	4.714	2.147	1.578	2.431	2.800	1.783
10	Std. Deviation	2.166	8.030	4.594	3.183	1.456	4.151	2.734	2.235
11	Std. Error of Mean	0.3829	0.9335	0.8681	0.5458	0.1820	0.5451	0.4992	0.4661
12									
13	Lower 95% CI of mean	0.4377	6.437	2.933	1.037	1.214	1.340	1.779	0.8160
14	Upper 95% CI of mean	2.000	10.16	6.495	3.258	1.942	3.523	3.821	2.749
15									
16	D'Agostino & Pearson omnibus normality test								
17	K2	36.49	15.44	16.39	58.86	8.945	58.96	47.55	9.159
18	P value	< 0.0001	0.0004	0.0003	< 0.0001	0.0114	< 0.0001	< 0.0001	0.0103
19	Passed normality test (alpha=0.05)?	No	No	No	No	No	No	No	No
20	P value summary	****	***	***	****	*	****	****	*
21									
22	Sum	39.00	614.0	132.0	73.00	101.0	141.0	84.00	41.00

1way ANOVA
ANOVA

1	Table Analyzed	53BP 1
2		
3	Kruskal-Wallis test	
4	P value	< 0.0001
5	Exact or approximate P value?	Approximate
6	P value summary	****
7	Do the medians vary signif. (P < 0.05)	Yes
8	Number of groups	8
9	Kruskal-Wallis statistic	58.08
10		
11	Data summary	
12	Number of treatments (columns)	8
13	Number of values (total)	343

**1way ANOVA** Multiple comparisons						
**1**	Number of families	1				
**2**	Number of comparisons per family	7				
**3**	Alpha	0.05				
**4**						
**5**	Dunn's multiple comparisons test	Mean rank diff.	Significant?	Summary		
**6**						
**7**	Non-irradiated vs. PTV	-118.8	Yes	****		
**8**	Non-irradiated vs. -5 to +20mm	-105.0	Yes	***		
**9**	Non-irradiated vs. +45 to +60mm	-44.02	No	ns		
**10**	Non-irradiated vs. +87 to +112mm	-35.70	No	ns		
**11**	Non-irradiated vs. +162 to +187mm	-33.91	No	ns		
**12**	Non-irradiated vs. +87 to +112mm	-83.58	Yes	**		
**13**	Non-irradiated vs. +162 to +187mm	-29.87	No	ns		
**14**						
**15**						
**16**	Test details	Mean rank 1	Mean rank 2	Mean rank diff.	n1	n2
**17**						
**18**	Non-irradiated vs. PTV	111.7	230.6	-118.8	32	74
**19**	Non-irradiated vs. -5 to +20mm	111.7	216.7	-105.0	32	28
**20**	Non-irradiated vs. +45 to +60mm	111.7	155.7	-44.02	32	34
**21**	Non-irradiated vs. +87 to +112mm	111.7	147.4	-35.70	32	64
**22**	Non-irradiated vs. +162 to +187mm	111.7	145.6	-33.91	32	58
**23**	Non-irradiated vs. +87 to +112mm	111.7	195.3	-83.58	32	30
**24**	Non-irradiated vs. +162 to +187mm	111.7	141.6	-29.87	32	23
