# Supplementary material for: The out-of-field dose in radiation therapy induces delayed tumorigenesis by senescence evasion
Source: eLife. 2022 Mar 18;11:e67190. doi: 10.7554/eLife.67190 (PMC8933005; doi:10.7554/eLife.67190)
Supplement: Figure 4—source data 2. [file elife-67190-fig4-data2.pdf]

Col. stats		A	B	C	D
		Non-irradiated	Total body	0 to +10	+10 to +20
		Y	Y	Y	Y
1	Number of values	455	469	1635	1186
2					
3	Minimum	0.0	0.0	0.0	0.0
4	25% Percentile	0.0	0.0	0.0	0.0
5	Median	0.0	2.000	1.000	3.000
6	75% Percentile	2.000	10.00	11.00	14.00
7	Maximum	178.0	186.0	398.0	432.0
8					
9	Mean	4.237	9.838	12.04	14.13
10	Std. Deviation	13.97	20.39	30.52	30.21
11	Std. Error of Mean	0.6551	0.9413	0.7549	0.8772
12					
13	Lower 95% CI of mean	2.950	7.988	10.56	12.41
14	Upper 95% CI of mean	5.525	11.69	13.52	15.86
15					
16	D'Agostino & Pearson omnibus normality test				
17	K2	635.9	424.3	1962	1415
18	P value	< 0.0001	< 0.0001	< 0.0001	< 0.0001
19	Passed normality test (alpha=0.05)?	No	No	No	No
20	P value summary	****	****	****	****
21					
22	Sum	1928	4614	19689	16763

1way ANOVA<br>ANOVA		
1	Table Analyzed	XRCC1 derme data pool
2		
3	Kruskal-Wallis test	
4	P value	< 0.0001
5	Exact or approximate P value?	Approximate
6	P value summary	****
7	Do the medians vary signif. (P < 0.05)	Yes
8	Number of groups	4
9	Kruskal-Wallis statistic	120.4
10		
11	Data summary	
12	Number of treatments (columns)	4
13	Number of values (total)	3745

1way ANOVA<br>Multiple comparisons						
1	Number of families	1				
2	Number of comparisons per family	3				
3	Alpha	0.05				
4						
5	Dunn's multiple comparisons test	Mean rank diff.	Significant?	Summary		
6						
7	Non-irradiated vs. Total body	-531.5	Yes	****		
8	Non-irradiated vs. 0 to +10	-460.5	Yes	****		
9	Non-irradiated vs. +10 to +20	-704.6	Yes	****		
10						
11						
12	Test details	Mean rank 1	Mean rank 2	Mean rank diff.	n1	n2
13						
14	Non-irradiated vs. Total body	1382	1914	-531.5	455	469
15	Non-irradiated vs. 0 to +10	1382	1843	-460.5	455	1635
16	Non-irradiated vs. +10 to +20	1382	2087	-704.6	455	1186
