# Supplementary material for: The out-of-field dose in radiation therapy induces delayed tumorigenesis by senescence evasion
Source: eLife. 2022 Mar 18;11:e67190. doi: 10.7554/eLife.67190 (PMC8933005; doi:10.7554/eLife.67190)
Supplement: Figure 4—source data 4. [file elife-67190-fig4-data4.pdf]

| Col. stats | | A | B | C | D |
|---|---|---|---|---|---|
| | | Non-irradiated | Total body | 0 to +10 | +10 to +20 |
| | | Y | Y | Y | Y |
| 1 | Number of values | 579 | 376 | 3138 | 1457 |
| 2 | | | | | |
| 3 | Minimum | 0.0 | 0.0 | 0.0 | 0.0 |
| 4 | 25% Percentile | 0.0 | 0.0 | 0.0 | 0.0 |
| 5 | Median | 0.0 | 1.000 | 0.0 | 0.0 |
| 6 | 75% Percentile | 0.0 | 6.000 | 0.0 | 0.0 |
| 7 | Maximum | 33.00 | 51.00 | 44.00 | 51.00 |
| 8 | | | | | |
| 9 | Mean | 0.3765 | 4.856 | 0.3442 | 0.2841 |
| 10 | Std. Deviation | 2.145 | 8.453 | 1.561 | 2.089 |
| 11 | Std. Error of Mean | 0.08915 | 0.4359 | 0.02787 | 0.05472 |
| 12 | | | | | |
| 13 | Lower 95% CI of mean | 0.2014 | 3.999 | 0.2895 | 0.1768 |
| 14 | Upper 95% CI of mean | 0.5516 | 5.714 | 0.3988 | 0.3915 |
| 15 | | | | | |
| 16 | D'Agostino & Pearson omnibus normality test | | | | |
| 17 | K2 | 950.7 | 223.2 | 5511 | 2908 |
| 18 | P value | < 0.0001 | < 0.0001 | < 0.0001 | < 0.0001 |
| 19 | Passed normality test (alpha=0.05)? | No | No | No | No |
| 20 | P value summary | **** | **** | **** | **** |
| 21 | | | | | |
| 22 | Sum | 218.0 | 1826 | 1080 | 414.0 |

| 1way ANOVA<br>ANOVA | | |
|---|---|---|
| | | |
| 1 | Table Analyzed | Data 1 |
| 2 | | |
| 3 | Kruskal-Wallis test | |
| 4 | P value | < 0.0001 |
| 5 | Exact or approximate P value? | Approximate |
| 6 | P value summary | **** |
| 7 | Do the medians vary signif. (P < 0.05) | Yes |
| 8 | Number of groups | 4 |
| 9 | Kruskal-Wallis statistic | -423.7 |
| 10 | | |
| 11 | Data summary | |
| 12 | Number of treatments (columns) | 4 |
| 13 | Number of values (total) | 5550 |

**1way ANOVA**
Multiple comparisons

| | | | | | | |
|---|---|---|---|---|---|---|
| 1 | Number of families | 1 | | | | |
| 2 | Number of comparisons per family | 3 | | | | |
| 3 | Alpha | 0.05 | | | | |
| 4 | | | | | | |
| 5 | Dunn's multiple comparisons test | Mean rank diff. | Significant? | Summary | | |
| 6 | | | | | | |
| 7 | Non-irradiated vs. Total body | -1315 | Yes | **** | | |
| 8 | Non-irradiated vs. 0 to +10 | -72.63 | No | ns | | |
| 9 | Non-irradiated vs. +10 to +20 | 84.12 | No | ns | | |
| 10 | | | | | | |
| 11 | | | | | | |
| 12 | Test details | Mean rank 1 | Mean rank 2 | Mean rank diff. | n1 | n2 |
| 13 | | | | | | |
| 14 | Non-irradiated vs. Total body | 2667 | 3982 | -1315 | 579 | 376 |
| 15 | Non-irradiated vs. 0 to +10 | 2667 | 2740 | -72.63 | 579 | 3138 |
| 16 | Non-irradiated vs. +10 to +20 | 2667 | 2583 | 84.12 | 579 | 1457 |
