# Supplementary material for: The out-of-field dose in radiation therapy induces delayed tumorigenesis by senescence evasion
Source: eLife. 2022 Mar 18;11:e67190. doi: 10.7554/eLife.67190 (PMC8933005; doi:10.7554/eLife.67190)
Supplement: Figure 4—figure supplement 1—source data 2. [file elife-67190-fig4-figsupp1-data2.pdf]

Col. stats		A	B	C	D
		Non-irradiated	Total body	0 to +10	+10 to +20
		Y	Y	Y	Y
1	Number of values	688	835	1563	1285
2					
3	Minimum	0.0	0.0	0.0	0.0
4	25% Percentile	0.0	0.0	0.0	0.0
5	Median	0.0	2.000	0.0	2.000
6	75% Percentile	1.000	14.00	3.000	23.00
7	Maximum	188.0	874.0	408.0	700.0
8					
9	Mean	3.529	15.70	8.474	22.61
10	Std. Deviation	14.68	44.68	28.59	53.86
11	Std. Error of Mean	0.5597	1.546	0.7230	1.502
12					
13	Lower 95% CI of mean	2.430	12.67	7.056	19.66
14	Upper 95% CI of mean	4.628	18.74	9.892	25.55
15					
16	D'Agostino & Pearson omnibus normality test				
17	K2	970.7	1421	2025	1536
18	P value	< 0.0001	< 0.0001	< 0.0001	< 0.0001
19	Passed normality test (alpha=0.05)?	No	No	No	No
20	P value summary	****	****	****	****
21					
22	Sum	2428	13113	13245	29049

1way ANOVA<br>ANOVA		
**1**	Table Analyzed	Data 1
**2**		
**3**	Kruskal-Wallis test	
**4**	P value	< 0.0001
**5**	Exact or approximate P value?	Approximate
**6**	P value summary	****
**7**	Do the medians vary signif. (P < 0.05)	Yes
**8**	Number of groups	4
**9**	Kruskal-Wallis statistic	286.0
**10**		
**11**	Data summary	
**12**	Number of treatments (columns)	4
**13**	Number of values (total)	4371

1way ANOVA
Multiple comparisons

1	Number of families	1				
2	Number of comparisons per family	3				
3	Alpha	0.05				
4						
5	Dunn's multiple comparisons test	Mean rank diff.	Significant?	Summary		
6						
7	Non-irradiated vs. Total body	-806.7	Yes	****		
8	Non-irradiated vs. 0 to +10	-295.0	Yes	****		
9	Non-irradiated vs. +10 to +20	-888.5	Yes	****		
10						
11						
12	Test details	Mean rank 1	Mean rank 2	Mean rank diff.	n1	n2
13						
14	Non-irradiated vs. Total body	1665	2472	-806.7	688	835
15	Non-irradiated vs. 0 to +10	1665	1960	-295.0	688	1563
16	Non-irradiated vs. +10 to +20	1665	2554	-888.5	688	1285
