# Supplementary material for: The out-of-field dose in radiation therapy induces delayed tumorigenesis by senescence evasion
Source: eLife. 2022 Mar 18;11:e67190. doi: 10.7554/eLife.67190 (PMC8933005; doi:10.7554/eLife.67190)
Supplement: Figure 5—source data 2. [file elife-67190-fig5-data2.pdf]

Col. stats		A	B	C
		Non irradiated	1x2 Gy	5x2 Gy
		Y	Y	Y
1	Number of values	185	179	181
2				
3	Minimum	0.0	0.0	0.0
4	25% Percentile	0.0	0.0	0.0
5	Median	0.0	0.0	0.0
6	75% Percentile	0.0	49.07	27.49
7	Maximum	146.8	105.2	132.0
8				
9	Mean	12.39	22.61	16.59
10	Std. Deviation	30.23	30.10	26.53
11	Std. Error of Mean	2.223	2.250	1.972
12				
13	Lower 95% CI of mean	8.006	18.17	12.70
14	Upper 95% CI of mean	16.78	27.05	20.48
15				
16	D'Agostino & Pearson omnibus normality test			
17	K2	96.88	22.13	84.41
18	P value	< 0.0001	< 0.0001	< 0.0001
19	Passed normality test (alpha=0.05)?	No	No	No
20	P value summary	****	****	****
21				
22	Sum	2292	4048	3004

1way ANOVA
ANOVA

1	Table Analyzed	PTV pH8
2		
3	Kruskal-Wallis test	
4	P value	< 0.0001
5	Exact or approximate P value?	Approximate
6	P value summary	****
7	Do the medians vary signif. (P < 0.05)	Yes
8	Number of groups	3
9	Kruskal-Wallis statistic	23.17
10		
11	Data summary	
12	Number of treatments (columns)	3
13	Number of values (total)	545

**1way ANOVA** Multiple comparisons						
**1**	Number of families	1				
**2**	Number of comparisons per family	3				
**3**	Alpha	0.05				
**4**						
**5**	Dunn's multiple comparisons test	Mean rank diff.	Significant?	Summary		
**6**						
**7**	Non irradiated vs. 1x2 Gy	-63.11	Yes	****		
**8**	Non irradiated vs. 5x2 Gy	-46.66	Yes	**		
**9**	1x2 Gy vs. 5x2 Gy	16.45	No	ns		
**10**						
**11**						
**12**	Test details	Mean rank 1	Mean rank 2	Mean rank diff.	n1	n2
**13**						
**14**	Non irradiated vs. 1x2 Gy	236.8	299.9	-63.11	185	179
**15**	Non irradiated vs. 5x2 Gy	236.8	283.4	-46.66	185	181
**16**	1x2 Gy vs. 5x2 Gy	299.9	283.4	16.45	179	181

Col. stats		A	B	C
		Non irradiated	1x2 Gy	5x2 Gy
		Y	Y	Y
1	Number of values	300	255	167
2				
3	Minimum	0.0	0.0	0.0
4	25% Percentile	0.0	0.0	0.0
5	Median	0.0	0.0	0.0
6	75% Percentile	0.0	0.0	0.0
7	Maximum	152.6	67.91	149.8
8				
9	Mean	4.912	3.955	4.857
10	Std. Deviation	18.04	12.07	18.83
11	Std. Error of Mean	1.042	0.7558	1.457
12				
13	Lower 95% CI of mean	2.862	2.467	1.981
14	Upper 95% CI of mean	6.961	5.444	7.734
15				
16	D'Agostino & Pearson omnibus normality test			
17	K2	314.5	187.1	195.8
18	P value	< 0.0001	< 0.0001	< 0.0001
19	Passed normality test (alpha=0.05)?	No	No	No
20	P value summary	****	****	****
21				
22	Sum	1473	1009	811.2

1way ANOVA<br>ANOVA		
1	Table Analyzed	margin from - 5 mm to 20 mm
2		
3	Kruskal-Wallis test	
4	P value	0.6494
5	Exact or approximate P value?	Approximate
6	P value summary	ns
7	Do the medians vary signif. (P < 0.05)	No
8	Number of groups	3
9	Kruskal-Wallis statistic	0.8634
10		
11	Data summary	
12	Number of treatments (columns)	3
13	Number of values (total)	722

1way ANOVA<br>Multiple comparisons						
1	Number of families	1				
2	Number of comparisons per family	3				
3	Alpha	0.05				
4						
5	Dunn's multiple comparisons test	Mean rank diff.	Significant?	Summary		
6						
7	Non irradiated vs. 1x2 Gy	-5.617	No	ns		
8	Non irradiated vs. 5x2 Gy	3.842	No	ns		
9	1x2 Gy vs. 5x2 Gy	9.459	No	ns		
10						
11						
12	Test details	Mean rank 1	Mean rank 2	Mean rank diff.	n1	n2
13						
14	Non irradiated vs. 1x2 Gy	360.4	366.0	-5.617	300	255
15	Non irradiated vs. 5x2 Gy	360.4	356.6	3.842	300	167
16	1x2 Gy vs. 5x2 Gy	366.0	356.6	9.459	255	167

Col. stats		A	B	C
		Non irradiated	1x2 Gy	5x2 Gy
		Y	Y	Y
1	Number of values	232	239	247
2				
3	Minimum	0.0	0.0	0.0
4	25% Percentile	0.0	0.0	0.0
5	Median	0.0	0.0	0.0
6	75% Percentile	0.0	40.79	0.0
7	Maximum	446.2	195.3	179.4
8				
9	Mean	19.52	20.53	19.79
10	Std. Deviation	46.23	36.47	38.29
11	Std. Error of Mean	3.035	2.359	2.437
12				
13	Lower 95% CI of mean	13.54	15.89	14.99
14	Upper 95% CI of mean	25.50	25.18	24.59
15				
16	D'Agostino & Pearson omnibus normality test			
17	K2	253.9	98.48	82.48
18	P value	< 0.0001	< 0.0001	< 0.0001
19	Passed normality test (alpha=0.05)?	No	No	No
20	P value summary	****	****	****
21				
22	Sum	4530	4908	4889

1way ANOVA
ANOVA

1	Table Analyzed	margin from 22 mm to 47 mm
2		
3	Kruskal-Wallis test	
4	P value	0.4263
5	Exact or approximate P value?	Approximate
6	P value summary	ns
7	Do the medians vary signif. (P < 0.05)	No
8	Number of groups	3
9	Kruskal-Wallis statistic	1.705
10		
11	Data summary	
12	Number of treatments (columns)	3
13	Number of values (total)	718

1way ANOVA
Multiple comparisons

1	Number of families	1				
2	Number of comparisons per family	3				
3	Alpha	0.05				
4						
5	Dunn's multiple comparisons test	Mean rank diff.	Significant?	Summary		
6						
7	Non irradiated vs. 1x2 Gy	-18.63	No	ns		
8	Non irradiated vs. 5x2 Gy	-5.646	No	ns		
9	1x2 Gy vs. 5x2 Gy	12.98	No	ns		
10						
11						
12	Test details	Mean rank 1	Mean rank 2	Mean rank diff.	n1	n2
13						
14	Non irradiated vs. 1x2 Gy	351.4	370.0	-18.63	232	239
15	Non irradiated vs. 5x2 Gy	351.4	357.0	-5.646	232	247
16	1x2 Gy vs. 5x2 Gy	370.0	357.0	12.98	239	247
