# Supplementary material for: The out-of-field dose in radiation therapy induces delayed tumorigenesis by senescence evasion
Source: eLife. 2022 Mar 18;11:e67190. doi: 10.7554/eLife.67190 (PMC8933005; doi:10.7554/eLife.67190)
Supplement: Figure 5—source data 3. [file elife-67190-fig5-data3.pdf]

Col. stats		A	B	C
		Non irradiated	1x2 Gy	5x2 Gy
		Y	Y	Y
1	Number of values	46	43	52
2				
3	Minimum	68.12	72.92	34.74
4	25% Percentile	114.4	120.3	80.23
5	Median	143.0	149.7	98.34
6	75% Percentile	165.6	191.0	121.8
7	Maximum	221.7	265.1	201.7
8				
9	Mean	138.7	158.2	105.7
10	Std. Deviation	37.21	49.67	34.70
11	Std. Error of Mean	5.487	7.575	4.812
12				
13	Lower 95% CI of mean	127.7	142.9	96.00
14	Upper 95% CI of mean	149.8	173.5	115.3
15				
16	D'Agostino & Pearson omnibus normality test			
17	K2	0.7604	2.164	7.984
18	P value	0.6837	0.3390	0.0185
19	Passed normality test (alpha=0.05)?	Yes	Yes	No
20	P value summary	ns	ns	*
21				
22	Sum	6380	6802	5494

1way ANOVA<br>ANOVA		
1	Table Analyzed	ph12 PTV
2		
3	Kruskal-Wallis test	
4	P value	< 0.0001
5	Exact or approximate P value?	Approximate
6	P value summary	****
7	Do the medians vary signif. (P < 0.05)	Yes
8	Number of groups	3
9	Kruskal-Wallis statistic	31.90
10		
11	Data summary	
12	Number of treatments (columns)	3
13	Number of values (total)	141

1way ANOVA
Multiple comparisons

1	Number of families	1				
2	Number of comparisons per family	3				
3	Alpha	0.05				
4						
5	Dunn's multiple comparisons test	Mean rank diff.	Significant?	Summary		
6						
7	Non irradiated vs. 1x2 Gy	-12.06	No	ns		
8	Non irradiated vs. 5x2 Gy	33.20	Yes	***		
9	1x2 Gy vs. 5x2 Gy	45.26	Yes	****		
10						
11						
12	Test details	Mean rank 1	Mean rank 2	Mean rank diff.	n1	n2
13						
14	Non irradiated vs. 1x2 Gy	79.57	91.63	-12.06	46	43
15	Non irradiated vs. 5x2 Gy	79.57	46.37	33.20	46	52
16	1x2 Gy vs. 5x2 Gy	91.63	46.37	45.26	43	52

Col. stats		A	B	C
		Non irradiated	1x2 Gy	5x2 Gy
		Y	Y	Y
1	Number of values	80	55	80
2				
3	Minimum	64.63	92.49	110.7
4	25% Percentile	99.05	129.0	143.9
5	Median	121.5	148.5	174.2
6	75% Percentile	144.0	164.7	188.1
7	Maximum	178.5	224.7	230.4
8				
9	Mean	119.6	149.0	168.5
10	Std. Deviation	27.49	27.70	31.13
11	Std. Error of Mean	3.073	3.736	3.481
12				
13	Lower 95% CI of mean	113.5	141.5	161.5
14	Upper 95% CI of mean	125.7	156.5	175.4
15				
16	D'Agostino & Pearson omnibus normality test			
17	K2	1.641	3.487	5.124
18	P value	0.4402	0.1749	0.0771
19	Passed normality test (alpha=0.05)?	Yes	Yes	Yes
20	P value summary	ns	ns	ns
21				
22	Sum	9569	8193	13478

1way ANOVA<br>ANOVA					
1	Table Analyzed	ph12 margin from -5mm to 20 mm			
2					
3	ANOVA summary				
4	F	57.58			
5	P value	< 0.0001			
6	P value summary	****			
7	Are differences among means statistically significant? (P < 0.05)	Yes			
8	R square	0.3520			
9					
10	Brown-Forsythe test				
11	F (DFn, DFd)	1.062 (2, 212)			
12	P value	0.3474			
13	P value summary	ns			
14	Significantly different standard deviations? (P < 0.05)	No			
15					
16	Bartlett's test				
17	Bartlett's statistic (corrected)	1.480			
18	P value	0.4772			
19	P value summary	ns			
20	Significantly different standard deviations? (P < 0.05)	No			
21					
22	ANOVA table	SS	DF	MS	F (DFn, DFd)
23	Treatment (between columns)	96526	2	48263	F (2, 212) = 57.58
24	Residual (within columns)	177701	212	838.2	
25	Total	274227	214		
26					
27	Data summary				
28	Number of treatments (columns)	3			
29	Number of values (total)	215			

**1**	
**2**	
**3**	
**4**	
**5**	
**6**	
**7**	
**8**	
**9**	
**10**	
**11**	
**12**	
**13**	
**14**	
**15**	
**16**	
**17**	
**18**	
**19**	
**20**	
**21**	
**22**	P value
**23**	P < 0.0001
**24**	
**25**	
**26**	
**27**	
**28**	
**29**	

1way ANOVA
Multiple comparisons

**1**	Number of families	1							
**2**	Number of comparisons per family	3							
**3**	Alpha	0.05							
**4**									
**5**	Tukey's multiple comparisons test	Mean Diff.	95% CI of diff.	Significant?	Summary				
**6**									
**7**	Non irradiated vs. 1x2 Gy	-29.37	-41.34 to -17.40	Yes	****				
**8**	Non irradiated vs. 5x2 Gy	-48.87	-59.67 to -38.07	Yes	****				
**9**	1x2 Gy vs. 5x2 Gy	-19.50	-31.47 to -7.535	Yes	***				
**10**									
**11**									
**12**	Test details	Mean 1	Mean 2	Mean Diff.	SE of diff.	n1	n2	q	DF
**13**									
**14**	Non irradiated vs. 1x2 Gy	119.6	149.0	-29.37	5.071	80	55	8.189	212
**15**	Non irradiated vs. 5x2 Gy	119.6	168.5	-48.87	4.578	80	80	15.10	212
**16**	1x2 Gy vs. 5x2 Gy	149.0	168.5	-19.50	5.071	55	80	5.439	212

Col. stats		A	B	C
		Non irradiated	1x2 Gy	5x2 Gy
		Y	Y	Y
1	Number of values	70	73	81
2				
3	Minimum	58.65	65.95	78.71
4	25% Percentile	92.76	109.9	115.8
5	Median	117.0	127.3	145.9
6	75% Percentile	141.5	152.6	172.1
7	Maximum	199.7	222.5	236.1
8				
9	Mean	119.1	132.4	147.4
10	Std. Deviation	33.90	34.31	36.48
11	Std. Error of Mean	4.051	4.016	4.053
12				
13	Lower 95% CI of mean	111.0	124.4	139.3
14	Upper 95% CI of mean	127.2	140.4	155.5
15				
16	D'Agostino & Pearson omnibus normality test			
17	K2	3.725	6.881	3.267
18	P value	0.1553	0.0321	0.1952
19	Passed normality test (alpha=0.05)?	Yes	No	Yes
20	P value summary	ns	*	ns
21				
22	Sum	8338	9667	11938

1way ANOVA<br>ANOVA		
**1**	Table Analyzed	ph12 margin from 22 mm to 47 mm
**2**		
**3**	Kruskal-Wallis test	
**4**	P value	< 0.0001
**5**	Exact or approximate P value?	Approximate
**6**	P value summary	****
**7**	Do the medians vary signif. (P < 0.05)	Yes
**8**	Number of groups	3
**9**	Kruskal-Wallis statistic	21.53
**10**		
**11**	Data summary	
**12**	Number of treatments (columns)	3
**13**	Number of values (total)	224

1way ANOVA<br>Multiple comparisons						
1	Number of families	1				
2	Number of comparisons per family	3				
3	Alpha	0.05				
4						
5	Dunn's multiple comparisons test	Mean rank diff.	Significant?	Summary		
6						
7	Non irradiated vs. 1x2 Gy	-23.56	No	ns		
8	Non irradiated vs. 5x2 Gy	-48.97	Yes	****		
9	1x2 Gy vs. 5x2 Gy	-25.42	Yes	*		
10						
11						
12	Test details	Mean rank 1	Mean rank 2	Mean rank diff.	n1	n2
13						
14	Non irradiated vs. 1x2 Gy	87.11	110.7	-23.56	70	73
15	Non irradiated vs. 5x2 Gy	87.11	136.1	-48.97	70	81
16	1x2 Gy vs. 5x2 Gy	110.7	136.1	-25.42	73	81
