# Supplementary material for: The out-of-field dose in radiation therapy induces delayed tumorigenesis by senescence evasion
Source: eLife. 2022 Mar 18;11:e67190. doi: 10.7554/eLife.67190 (PMC8933005; doi:10.7554/eLife.67190)
Supplement: Figure 5—figure supplement 1—source data 2. [file elife-67190-fig5-figsupp1-data2.pdf]

1way ANOVA
ANOVA

**1**	Table Analyzed	1x2 gy + 0h
**2**		
**3**	Kruskal-Wallis test	
**4**	P value	< 0.0001
**5**	Exact or approximate P value?	Approximate
**6**	P value summary	****
**7**	Do the medians vary signif. (P < 0.05)	Yes
**8**	Number of groups	4
**9**	Kruskal-Wallis statistic	121.4
**10**		
**11**	Data summary	
**12**	Number of treatments (columns)	4
**13**	Number of values (total)	311

1way ANOVA<br>Multiple comparisons						
1	Number of families	1				
2	Number of comparisons per family	3				
3	Alpha	0.05				
4						
5	Dunn's multiple comparisons test	Mean rank diff.	Significant?	Summary		
6						
7	Non irradiated vs. PTV	-103.3	Yes	****		
8	Non irradiated vs. margin from -5 to 20 mm	-121.8	Yes	****		
9	Non irradiated vs. margin from 22 to 47 mm	-161.8	Yes	****		
10						
11						
12	Test details	Mean rank 1	Mean rank 2	Mean rank diff.	n1	n2
13						
14	Non irradiated vs. PTV	60.44	163.7	-103.3	73	77
15	Non irradiated vs. margin from -5 to 20 mm	60.44	182.2	-121.8	73	107
16	Non irradiated vs. margin from 22 to 47 mm	60.44	222.3	-161.8	73	54

1way ANOVA
ANOVA

1	Table Analyzed	1x2gy + 72h
2		
3	Kruskal-Wallis test	
4	P value	0.0013
5	Exact or approximate P value?	Approximate
6	P value summary	**
7	Do the medians vary signif. (P < 0.05)	Yes
8	Number of groups	4
9	Kruskal-Wallis statistic	15.71
10		
11	Data summary	
12	Number of treatments (columns)	4
13	Number of values (total)	258

1way ANOVA<br>Multiple comparisons						
1	Number of families	1				
2	Number of comparisons per family	3				
3	Alpha	0.05				
4						
5	Dunn's multiple comparisons test	Mean rank diff.	Significant?	Summary		
6						
7	Non irradiated vs. PTV	21.84	No	ns		
8	Non irradiated vs. margin from -5 to 20 mm	-9.127	No	ns		
9	Non irradiated vs. margin from 22 to 47 mm	-29.63	No	ns		
10						
11						
12	Test details	Mean rank 1	Mean rank 2	Mean rank diff.	n1	n2
13						
14	Non irradiated vs. PTV	123.3	101.5	21.84	66	55
15	Non irradiated vs. margin from -5 to 20 mm	123.3	132.5	-9.127	66	62
16	Non irradiated vs. margin from 22 to 47 mm	123.3	153.0	-29.63	66	75

**1way ANOVA**<br>ANOVA		
**1**	Table Analyzed	5x2gy + 0h
**2**		
**3**	Kruskal-Wallis test	
**4**	P value	< 0.0001
**5**	Exact or approximate P value?	Approximate
**6**	P value summary	****
**7**	Do the medians vary signif. (P < 0.05)	Yes
**8**	Number of groups	4
**9**	Kruskal-Wallis statistic	63.98
**10**		
**11**	Data summary	
**12**	Number of treatments (columns)	4
**13**	Number of values (total)	556

1way ANOVA
Multiple comparisons

1	Number of families	1				
2	Number of comparisons per family	3				
3	Alpha	0.05				
4						
5	Dunn's multiple comparisons test	Mean rank diff.	Significant?	Summary		
6						
7	Non irradiated vs. PTV	-79.91	Yes	****		
8	Non irradiated vs. margin from -5 to 20 mm	-117.2	Yes	****		
9	Non irradiated vs. margin from 22 to 47 mm	-115.2	Yes	****		
10						
11						
12	Test details	Mean rank 1	Mean rank 2	Mean rank diff.	n1	n2
13						
14	Non irradiated vs. PTV	213.6	293.5	-79.91	205	131
15	Non irradiated vs. margin from -5 to 20 mm	213.6	330.9	-117.2	205	128
16	Non irradiated vs. margin from 22 to 47 mm	213.6	328.8	-115.2	205	92

1way ANOVA
ANOVA

1	Table Analyzed	5x2gy + 72h
2		
3	Kruskal-Wallis test	
4	P value	< 0.0001
5	Exact or approximate P value?	Approximate
6	P value summary	****
7	Do the medians vary signif. (P < 0.05)	Yes
8	Number of groups	4
9	Kruskal-Wallis statistic	40.66
10		
11	Data summary	
12	Number of treatments (columns)	4
13	Number of values (total)	376

1way ANOVA
Multiple comparisons

1	Number of families	1				
2	Number of comparisons per family	3				
3	Alpha	0.05				
4						
5	Dunn's multiple comparisons test	Mean rank diff.	Significant?	Summary		
6						
7	Non irradiated vs. PTV	-73.06	Yes	****		
8	Non irradiated vs. margin from -5 to 20 mm	-80.54	Yes	****		
9	Non irradiated vs. margin from 22 to 47 mm	-48.01	Yes	**		
10						
11						
12	Test details	Mean rank 1	Mean rank 2	Mean rank diff.	n1	n2
13						
14	Non irradiated vs. PTV	144.9	218.0	-73.06	127	86
15	Non irradiated vs. margin from -5 to 20 mm	144.9	225.5	-80.54	127	70
16	Non irradiated vs. margin from 22 to 47 mm	144.9	192.9	-48.01	127	93

1way ANOVA
ANOVA

1	Table Analyzed	10x2gy+0h				
2						
3	ANOVA summary					
4	F	34.86				
5	P value	< 0.0001				
6	P value summary	****				
7	Are differences among means statistically significant? (P < 0.05)	Yes				
8	R square	0.2352				
9						
10	Brown-Forsythe test					
11	F (DFn, DFd)	32.03 (3, 340)				
12	P value	< 0.0001				
13	P value summary	****				
14	Significantly different standard deviations? (P < 0.05)	Yes				
15						
16	Bartlett's test					
17	Bartlett's statistic (corrected)	80.00				
18	P value	< 0.0001				
19	P value summary	****				
20	Significantly different standard deviations? (P < 0.05)	Yes				
21						
22	ANOVA table	SS	DF	MS	F (DFn, DFd)	P value
23	Treatment (between columns)	69281	3	23094	F (3, 340) = 34.86	P < 0.0001
24	Residual (within columns)	225245	340	662.5		
25	Total	294526	343			
26						
27	Data summary					
28	Number of treatments (columns)	4				
29	Number of values (total)	344				

1way ANOVA
Multiple comparisons

**1**	Number of families	1							
**2**	Number of comparisons per family	3							
**3**	Alpha	0.05							
**4**									
**5**	Dunnett's multiple comparisons test	Mean Diff.	95% CI of diff.	Significant?	Summary				
**6**									
**7**	Non irradiated vs. PTV	-39.16	-48.39 to -29.93	Yes	****				
**8**	Non irradiated vs. margin from -5 to 20 mm	-27.06	-36.34 to -17.79	Yes	****				
**9**	Non irradiated vs. margin from 22 to 47 mm	-26.31	-35.91 to -16.70	Yes	****				
**10**									
**11**									
**12**	Test details	Mean 1	Mean 2	Mean Diff.	SE of diff.	n1	n2	q	DF
**13**									
**14**	Non irradiated vs. PTV	46.61	85.77	-39.16	3.912	81	93	10.01	340
**15**	Non irradiated vs. margin from -5 to 20 mm	46.61	73.68	-27.06	3.932	81	91	6.883	340
**16**	Non irradiated vs. margin from 22 to 47 mm	46.61	72.92	-26.31	4.070	81	79	6.463	340

1way ANOVA
ANOVA

**1**	Table Analyzed	10x2gy + 72h
**2**		
**3**	Kruskal-Wallis test	
**4**	P value	0.4122
**5**	Exact or approximate P value?	Approximate
**6**	P value summary	ns
**7**	Do the medians vary signif. (P < 0.05)	No
**8**	Number of groups	3
**9**	Kruskal-Wallis statistic	1.773
**10**		
**11**	Data summary	
**12**	Number of treatments (columns)	3
**13**	Number of values (total)	362

1way ANOVA<br>Multiple comparisons						
1	Number of families	1				
2	Number of comparisons per family	2				
3	Alpha	0.05				
4						
5	Dunn's multiple comparisons test	Mean rank diff.	Significant?	Summary		
6						
7	Non irradiated vs. margin from -5 to 20 mm	-18.88	No	ns		
8	Non irradiated vs. margin from 22 to 47 mm	-11.46	No	ns		
9						
10						
11	Test details	Mean rank 1	Mean rank 2	Mean rank diff.	n1	n2
12						
13	Non irradiated vs. margin from -5 to 20 mm	170.0	188.8	-18.88	82	131
14	Non irradiated vs. margin from 22 to 47 mm	170.0	181.4	-11.46	82	149
