# Supplementary material for: The out-of-field dose in radiation therapy induces delayed tumorigenesis by senescence evasion
Source: eLife. 2022 Mar 18;11:e67190. doi: 10.7554/eLife.67190 (PMC8933005; doi:10.7554/eLife.67190)
Supplement: Figure 5—figure supplement 1—source data 4. [file elife-67190-fig5-figsupp1-data4.pdf]

1way ANOVA
ANOVA

1	Table Analyzed	1x2 gy + 0h
2		
3	Kruskal-Wallis test	
4	P value	< 0.0001
5	Exact or approximate P value?	Approximate
6	P value summary	****
7	Do the medians vary signif. (P < 0.05)	Yes
8	Number of groups	4
9	Kruskal-Wallis statistic	116.9
10		
11	Data summary	
12	Number of treatments (columns)	4
13	Number of values (total)	377

1way ANOVA<br>Multiple comparisons						
1	Number of families	1				
2	Number of comparisons per family	3				
3	Alpha	0.05				
4						
5	Dunn's multiple comparisons test	Mean rank diff.	Significant?	Summary		
6						
7	Non irradiated vs. PTV	-154.5	Yes	****		
8	Non irradiated vs. margin from -5 to 20 mm	-34.19	Yes	*		
9	Non irradiated vs. margin from 22 to 47 mm	-11.27	No	ns		
10						
11						
12	Test details	Mean rank 1	Mean rank 2	Mean rank diff.	n1	n2
13						
14	Non irradiated vs. PTV	150.3	304.8	-154.5	104	64
15	Non irradiated vs. margin from -5 to 20 mm	150.3	184.5	-34.19	104	102
16	Non irradiated vs. margin from 22 to 47 mm	150.3	161.6	-11.27	104	107

1way ANOVA
ANOVA

1	Table Analyzed	1x2gy + 72h
2		
3	Kruskal-Wallis test	
4	P value	0.1274
5	Exact or approximate P value?	Approximate
6	P value summary	ns
7	Do the medians vary signif. (P < 0.05)	No
8	Number of groups	4
9	Kruskal-Wallis statistic	5.695
10		
11	Data summary	
12	Number of treatments (columns)	4
13	Number of values (total)	399

1way ANOVA
Multiple comparisons

1	Number of families	1				
2	Number of comparisons per family	3				
3	Alpha	0.05				
4						
5	Dunn's multiple comparisons test	Mean rank diff.	Significant?	Summary		
6						
7	Non irradiated vs. PTV	-13.91	No	ns		
8	Non irradiated vs. margin from -5 to 20 mm	-23.97	No	ns		
9	Non irradiated vs. margin from 22 to 47 mm	-5.679	No	ns		
10						
11						
12	Test details	Mean rank 1	Mean rank 2	Mean rank diff.	n1	n2
13						
14	Non irradiated vs. PTV	188.7	202.6	-13.91	108	65
15	Non irradiated vs. margin from -5 to 20 mm	188.7	212.7	-23.97	108	127
16	Non irradiated vs. margin from 22 to 47 mm	188.7	194.4	-5.679	108	99

1way ANOVA<br>ANOVA		
1	Table Analyzed	5x2gy + 0h
2		
3	Kruskal-Wallis test	
4	P value	0.0046
5	Exact or approximate P value?	Approximate
6	P value summary	**
7	Do the medians vary signif. (P < 0.05)	Yes
8	Number of groups	4
9	Kruskal-Wallis statistic	13.04
10		
11	Data summary	
12	Number of treatments (columns)	4
13	Number of values (total)	573

1way ANOVA
Multiple comparisons

**1**	Number of families	1				
**2**	Number of comparisons per family	3				
**3**	Alpha	0.05				
**4**						
**5**	Dunn's multiple comparisons test	Mean rank diff.	Significant?	Summary		
**6**						
**7**	Non irradiated vs. PTV	-58.72	Yes	**		
**8**	Non irradiated vs. margin from -5 to 20 mm	-22.25	No	ns		
**9**	Non irradiated vs. margin from 22 to 47 mm	-34.58	No	ns		
**10**						
**11**						
**12**	Test details	Mean rank 1	Mean rank 2	Mean rank diff.	n1	n2
**13**						
**14**	Non irradiated vs. PTV	257.4	316.2	-58.72	137	142
**15**	Non irradiated vs. margin from -5 to 20 mm	257.4	279.7	-22.25	137	127
**16**	Non irradiated vs. margin from 22 to 47 mm	257.4	292.0	-34.58	137	167

1way ANOVA
ANOVA

1	Table Analyzed	5x2gy + 72h
2		
3	Kruskal-Wallis test	
4	P value	0.0325
5	Exact or approximate P value?	Approximate
6	P value summary	*
7	Do the medians vary signif. (P < 0.05)	Yes
8	Number of groups	4
9	Kruskal-Wallis statistic	8.771
10		
11	Data summary	
12	Number of treatments (columns)	4
13	Number of values (total)	378

**1way ANOVA**<br>Multiple comparisons						
**1**	Number of families	1				
**2**	Number of comparisons per family	3				
**3**	Alpha	0.05				
**4**						
**5**	Dunn's multiple comparisons test	Mean rank diff.	Significant?	Summary		
**6**						
**7**	Non irradiated vs. PTV	-8.869	No	ns		
**8**	Non irradiated vs. margin from -5 to 20 mm	13.82	No	ns		
**9**	Non irradiated vs. margin from 22 to 47 mm	17.49	No	ns		
**10**						
**11**						
**12**	Test details	Mean rank 1	Mean rank 2	Mean rank diff.	n1	n2
**13**						
**14**	Non irradiated vs. PTV	195.6	204.5	-8.869	93	89
**15**	Non irradiated vs. margin from -5 to 20 mm	195.6	181.8	13.82	93	92
**16**	Non irradiated vs. margin from 22 to 47 mm	195.6	178.1	17.49	93	104

1way ANOVA<br>ANOVA		
**1**	Table Analyzed	10x2gy+0h
**2**		
**3**	Kruskal-Wallis test	
**4**	P value	< 0.0001
**5**	Exact or approximate P value?	Approximate
**6**	P value summary	****
**7**	Do the medians vary signif. (P < 0.05)	Yes
**8**	Number of groups	4
**9**	Kruskal-Wallis statistic	32.86
**10**		
**11**	Data summary	
**12**	Number of treatments (columns)	4
**13**	Number of values (total)	424

1way ANOVA<br>Multiple comparisons						
1	Number of families	1				
2	Number of comparisons per family	3				
3	Alpha	0.05				
4						
5	Dunn's multiple comparisons test	Mean rank diff.	Significant?	Summary		
6						
7	Non irradiated vs. PTV	-51.50	Yes	**		
8	Non irradiated vs. margin from -5 to 20 mm	-0.3684	No	ns		
9	Non irradiated vs. margin from 22 to 47 mm	18.76	No	ns		
10						
11						
12	Test details	Mean rank 1	Mean rank 2	Mean rank diff.	n1	n2
13						
14	Non irradiated vs. PTV	206.5	258.0	-51.50	77	103
15	Non irradiated vs. margin from -5 to 20 mm	206.5	206.8	-0.3684	77	96
16	Non irradiated vs. margin from 22 to 47 mm	206.5	187.7	18.76	77	148

1way ANOVA
ANOVA

1	Table Analyzed	10x2gy + 72h
2		
3	Kruskal-Wallis test	
4	P value	0.3718
5	Exact or approximate P value?	Approximate
6	P value summary	ns
7	Do the medians vary signif. (P < 0.05)	No
8	Number of groups	3
9	Kruskal-Wallis statistic	1.979
10		
11	Data summary	
12	Number of treatments (columns)	3
13	Number of values (total)	337

1way ANOVA
Multiple comparisons

**1**	Number of families	1				
**2**	Number of comparisons per family	2				
**3**	Alpha	0.05				
**4**						
**5**	Dunn's multiple comparisons test	Mean rank diff.	Significant?	Summary		
**6**						
**7**	Non irradiated vs. margin from -5 to 20 mm	7.725	No	ns		
**8**	Non irradiated vs. margin from 22 to 47 mm	10.41	No	ns		
**9**						
**10**						
**11**	Test details	Mean rank 1	Mean rank 2	Mean rank diff.	n1	n2
**12**						
**13**	Non irradiated vs. margin from -5 to 20 mm	174.9	167.1	7.725	126	81
**14**	Non irradiated vs. margin from 22 to 47 mm	174.9	164.5	10.41	126	130
