# Supplementary material for: The out-of-field dose in radiation therapy induces delayed tumorigenesis by senescence evasion
Source: eLife. 2022 Mar 18;11:e67190. doi: 10.7554/eLife.67190 (PMC8933005; doi:10.7554/eLife.67190)
Supplement: Figure 5—figure supplement 3—source data 2. [file elife-67190-fig5-figsupp3-data2.pdf]

1way ANOVA
ANOVA

1	Table Analyzed	simplification H2O2 nuage
2		
3	Kruskal-Wallis test	
4	P value	< 0.0001
5	Exact or approximate P value?	Approximate
6	P value summary	****
7	Do the medians vary signif. (P < 0.05)	Yes
8	Number of groups	7
9	Kruskal-Wallis statistic	287.7
10		
11	Data summary	
12	Number of treatments (columns)	7
13	Number of values (total)	432

1way ANOVA
Multiple comparisons

1	Number of families	1				
2	Number of comparisons per family	6				
3	Alpha	0.05				
4						
5	Dunn's multiple comparisons test	Mean rank diff.	Significant?	Summary		
6						
7	Non-irradiated vs. 1x2Gy	-204.1	Yes	****		
8	Non-irradiated vs. 10x2Gy	-53.78	No	ns		
9	Non-irradiated vs. 1x2GY	-169.9	Yes	****		
10	Non-irradiated vs. 10x2Gy	-16.09	No	ns		
11	Non-irradiated vs. 1x2Gy	-219.2	Yes	****		
12	Non-irradiated vs. 10x2Gy	41.69	No	ns		
13						
14						
15	Test details	Mean rank 1	Mean rank 2	Mean rank diff.	n1	n2
16						
17	Non-irradiated vs. 1x2Gy	140.7	344.8	-204.1	64	33
18	Non-irradiated vs. 10x2Gy	140.7	194.5	-53.78	64	54
19	Non-irradiated vs. 1x2GY	140.7	310.6	-169.9	64	54
20	Non-irradiated vs. 10x2Gy	140.7	156.8	-16.09	64	57
21	Non-irradiated vs. 1x2Gy	140.7	359.9	-219.2	64	77
22	Non-irradiated vs. 10x2Gy	140.7	99.04	41.69	64	93

t test		
1	Table Analyzed	simplification H2O2 nuage
2		
3	Column C	10x2Gy
4	vs.	vs.
5	Column B	1x2Gy
6		
7	Unpaired t test	
8	P value	< 0.0001
9	P value summary	****
10	Significantly different? (P < 0.05)	Yes
11	One- or two-tailed P value?	Two-tailed
12	t, df	t=10.36 df=85
13		
14	How big is the difference?	
15	Mean ± SEM of column B	81.24 ± 5.840 N=33
16	Mean ± SEM of column C	28.27 ± 1.820 N=54
17	Difference between means	-52.97 ± 5.112
18	95% confidence interval	-63.13 to -42.80
19	R square	0.5581
20		
21	F test to compare variances	
22	F,DFn, Dfd	6.292, 32, 53
23	P value	< 0.0001
24	P value summary	****
25	Significantly different? (P < 0.05)	Yes

t test		
1	Table Analyzed	simplification H2O2 nuage
2		
3	Column E	10x2Gy
4	vs.	vs.
5	Column D	1x2GY
6		
7	Unpaired t test	
8	P value	< 0.0001
9	P value summary	****
10	Significantly different? (P < 0.05)	Yes
11	One- or two-tailed P value?	Two-tailed
12	t, df	t=10.84 df=109
13		
14	How big is the difference?	
15	Mean ± SEM of column D	62.03 ± 3.191 N=54
16	Mean ± SEM of column E	22.24 ± 1.905 N=57
17	Difference between means	-39.79 ± 3.671
18	95% confidence interval	-47.06 to -32.51
19	R square	0.5187
20		
21	F test to compare variances	
22	F,DFn, Dfd	2.658, 53, 56
23	P value	0.0004
24	P value summary	***
25	Significantly different? (P < 0.05)	Yes

t test		
1	Table Analyzed	simplification H2O2 nuage
2		
3	Column G	10x2Gy
4	vs.	vs.
5	Column F	1x2Gy
6		
7	Unpaired t test	
8	P value	< 0.0001
9	P value summary	****
10	Significantly different? (P < 0.05)	Yes
11	One- or two-tailed P value?	Two-tailed
12	t, df	t=20.80 df=168
13		
14	How big is the difference?	
15	Mean ± SEM of column F	89.07 ± 3.839 N=77
16	Mean ± SEM of column G	13.08 ± 0.9791 N=93
17	Difference between means	-75.99 ± 3.653
18	95% confidence interval	-83.20 to -68.78
19	R square	0.7204
20		
21	F test to compare variances	
22	F,DFn, Dfd	12.73, 76, 92
23	P value	< 0.0001
24	P value summary	****
25	Significantly different? (P < 0.05)	Yes
