# Supplementary figures and images for: The out-of-field dose in radiation therapy induces delayed tumorigenesis by senescence evasion
Source: eLife. 2022 Mar 18;11:e67190. doi: 10.7554/eLife.67190 (PMC8933005; doi:10.7554/eLife.67190)

## Slide 1
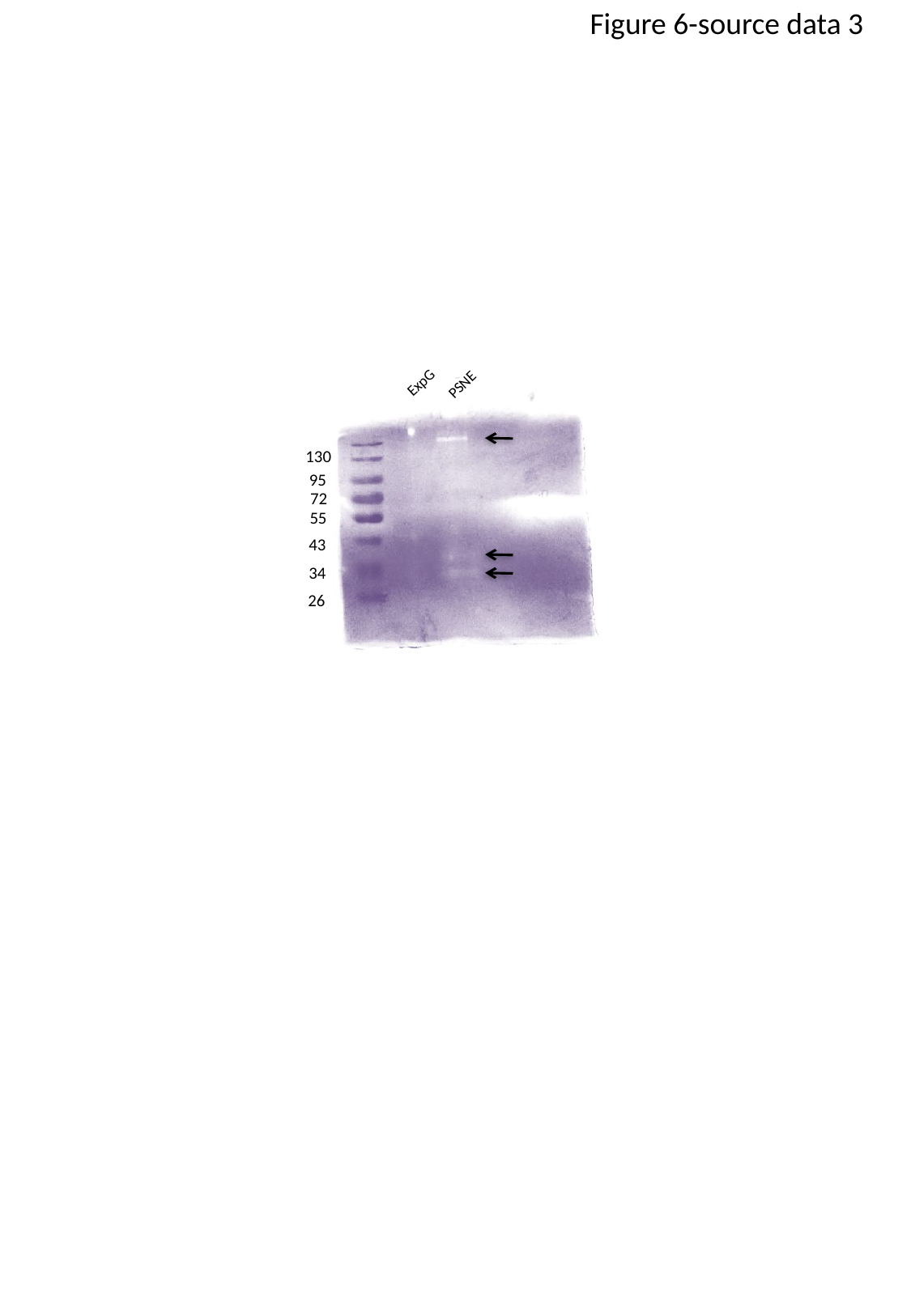

Figure 6-source data 3
ExpG
PSNE
150
130
95
72
55
43
34
26

Supplement: Figure 6—source data 3. [file elife-67190-fig6-data3.pptx]
