# Supplementary material for: The out-of-field dose in radiation therapy induces delayed tumorigenesis by senescence evasion
Source: eLife. 2022 Mar 18;11:e67190. doi: 10.7554/eLife.67190 (PMC8933005; doi:10.7554/eLife.67190)
Supplement: Figure 7—figure supplement 1—source data 1. [file elife-67190-fig7-figsupp1-data1.pptx]

## Slide 1
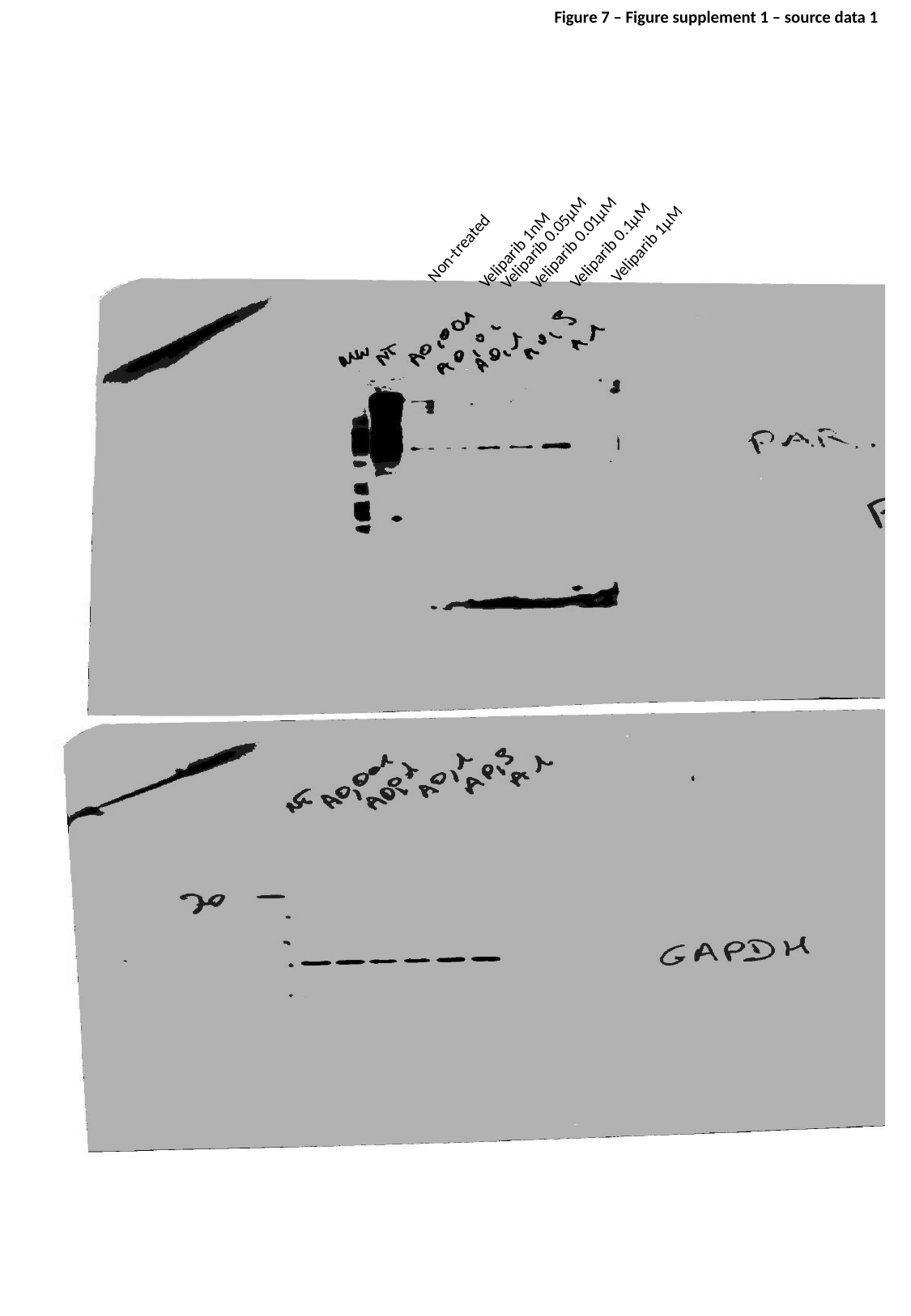

Figure 7 – Figure supplement 1 – source data 1
Veliparib 0.05µM
Veliparib 0.01µM
Veliparib 0.1µM
Veliparib 1µM
Veliparib 1nM
Non-treated
