# Supplementary material for: The out-of-field dose in radiation therapy induces delayed tumorigenesis by senescence evasion
Source: eLife. 2022 Mar 18;11:e67190. doi: 10.7554/eLife.67190 (PMC8933005; doi:10.7554/eLife.67190)
Supplement: Figure 7—figure supplement 4—source data 2. [file elife-67190-fig7-figsupp4-data2.pptx]

## Slide 1
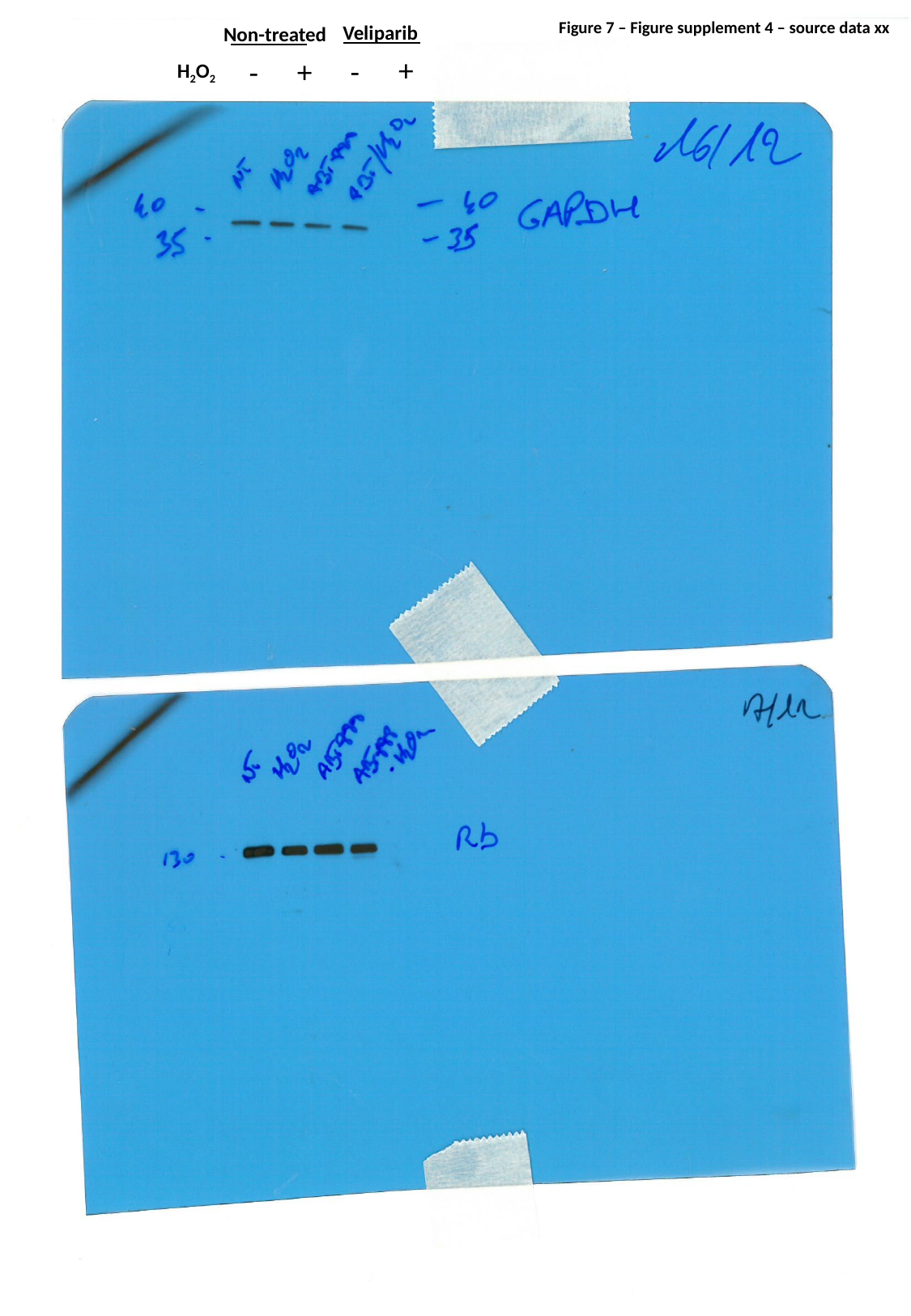

Figure 7 – Figure supplement 4 – source data xx
Veliparib
Non-treated
+
+
-
-
H2O2

## Slide 2
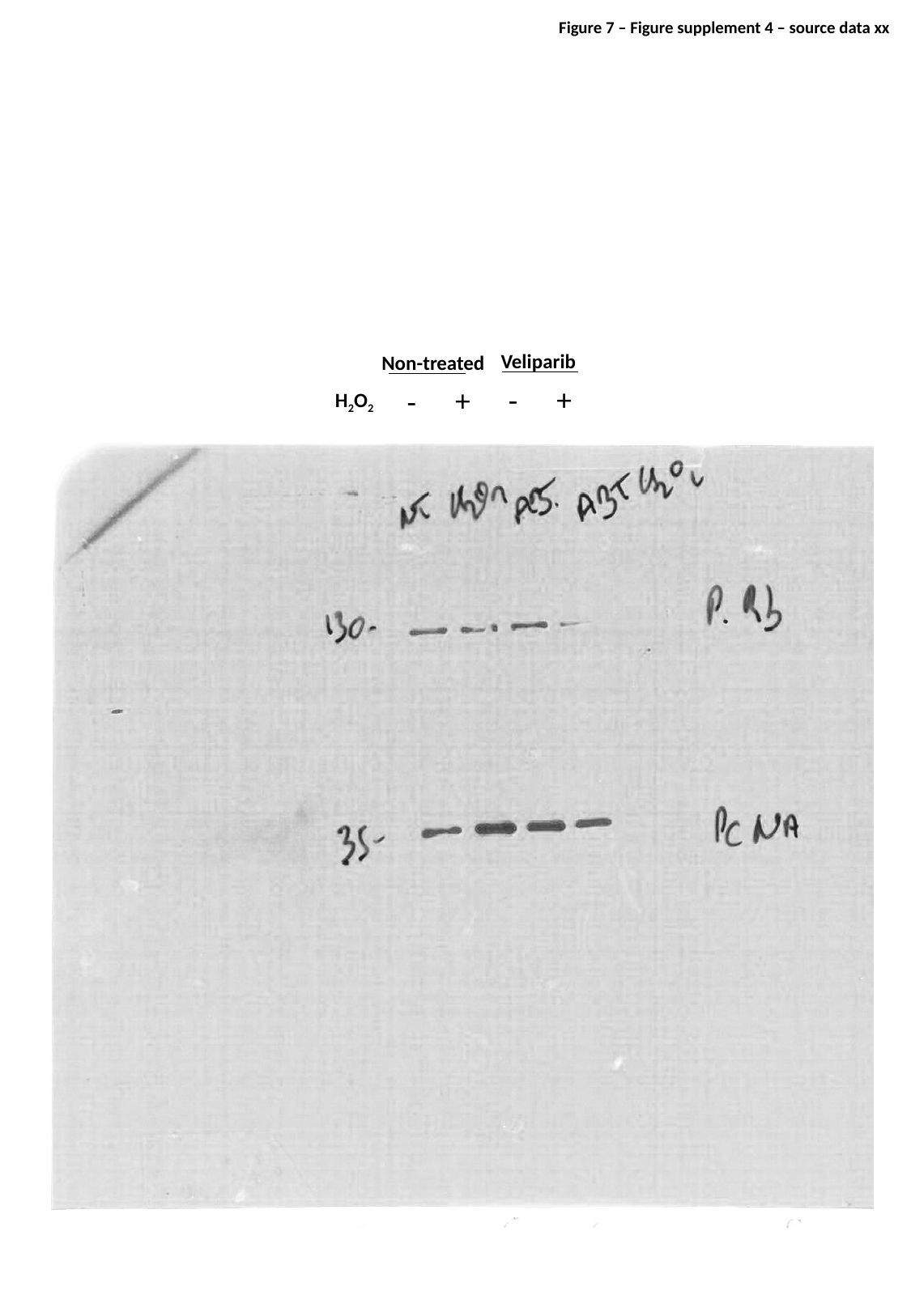

Figure 7 – Figure supplement 4 – source data xx
Veliparib
Non-treated
+
+
-
-
H2O2

## Slide 3
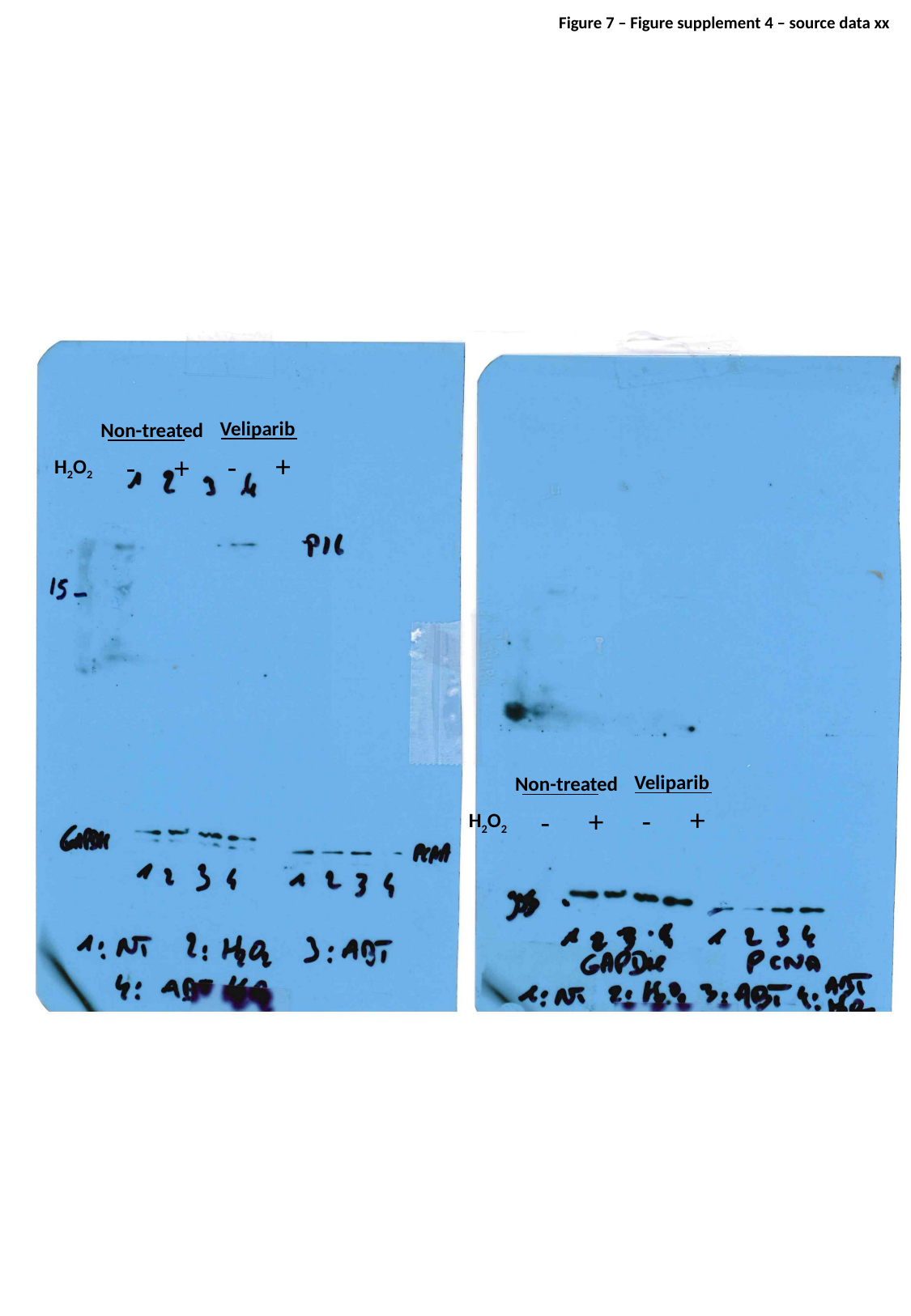

Figure 7 – Figure supplement 4 – source data xx
Veliparib
Non-treated
+
+
-
-
H2O2
Veliparib
Non-treated
+
+
-
-
H2O2
